# A Meta-Analysis and Systematic Review of the Efficacy of Twice Daily PPIs versus Once Daily for Treatment of Gastroesophageal Reflux Disease

**DOI:** 10.1155/2017/9865963

**Published:** 2017-08-22

**Authors:** Hongying Zhang, Zhiping Yang, Zhen Ni, Yongquan Shi

**Affiliations:** ^1^State Key Laboratory of Cancer Biology & Institute of Digestive Diseases, Xijing Hospital, Fourth Military Medical University, Xi'an, Shaanxi 710032, China; ^2^Department of Intensive Care Unit, Shaanxi Provincial People's Hospital, Xi'an, Shaanxi 710068, China

## Abstract

**Background:**

To investigate whether PPIs BID is superior to QD for treatment of GERD in a short time.

**Methods:**

We searched PubMed, Cochrane Library, Scopus, EMBASE, Ovid, EBSCO, and Web of Science databases (from 1998 to May 2016) to select RCTs, which compared the efficacy of PPIs BID versus QD for GERD. The primary outcomes were symptom relief or esophageal mucosal healing at weeks 4 and 8. The M-H method with fixed-effect or random-effect model was used to calculate RR and 95% CIs.

**Results:**

Seven RCTs were enrolled. The esophageal healing rates were higher in PPIs BID group (*P* = 0.01), and rabeprazole 20 mg BID can achieve better mucosal healing than 20 mg QD after 8 weeks (*P* < 0.05). However, no significant differences were observed in heartburn relief (*P* = 0.27), sustained symptom relief rates at week 4 (*P* = 0.05), 24 h pH monitoring after treatment (*P* = 0.11), endoscopic response at week 4 (*P* = 0.22), and adverse events (*P* = 0.18).

**Conclusion:**

PPIs BID more effectively improve endoscopic healing rate at week 8 than PPIs QD. But there are no significant differences in symptom relief, 24 h pH monitoring, sustained symptom relief, and endoscopic response at week 4.

## 1. Introduction

Gastroesophageal reflux disease (GERD) remains a prevalent disease worldwide, with East Asia showing prevalence estimates consistently below 10% [1]. The Montreal Definition of GERD states that GERD develops when the reflux of stomach contents causes troublesome symptoms and/or complications, which may be considered to be moderate-to-severe manifestations occurring on one or more days per week [2]. GERD patients may also have higher incidences of some subsequent complications such as esophageal adenocarcinoma, esophageal stricture, sleep disturbance, and some extra esophageal problems [3]. Previous study reported that the frequency of ambulatory visits in the United States for GERD increased significantly from 1995 to 2006 [4]. A breakdown of expenditure showed that direct medical costs of GERD were 65% and indirect costs were 19% of total disease-related expenditure [5]. Since the application of proton pump inhibitors (PPIs), the treatment and healing rates of reflux disease have improved significantly [6]. PPIs represent the first medical treatment choice for GERD, and in that, they are able to provide a 56–76% symptom relief [7] and 80–85% healing rates for esophageal lesions, which further reduce the incidence of complications [8]. However, it has been estimated that about 30% of GERD patients remain symptomatic on standard doses of PPIs once daily (QD) [9] and this group may be at increased risk of more serious complications including Barrett's esophagus [6]. For these patients who have an unsatisfactory response to PPIs QD, increasing to twice daily (BID) may be an alternative [10].

However, few studies have estimated the efficacy of PPIs BID for GERD treatment. In this meta-analysis, we investigated the effects of symptom relief, esophageal mucosa healing rates, 24 h pH monitoring results, and adverse events in patients with GERD after treatment with PPIs BID versus QD.

## 2. Methods

### 2.1. Searching Strategies

We performed a systematic search of databases from 1998 to May 2016. The search strategy consisted of a combination of the following MESH terms and text words: (gastroesophageal reflux disease, GERD, GORD, reflux esophagitis, RE, non-erosive reflux disease, NERD, Barrett's esophagus, BE, erosive esophagitis, and EE); (Proton Pump Inhibitors, PPIs, omeprazole, lansoprazole, pantoprazole, rabeprazole, and esomeprazole); and (twice daily). Titles and abstracts of English were screened for eligibility. The full text of selected trials was further reviewed independently by two independent investigators to confirm eligibility, assess quality, and extract data using excel. Bibliographies of all articles were reviewed to retrieve additional studies.

### 2.2. Inclusion Criteria

We included randomized controlled trials (RCTs) that aimed to investigate treatment efficacy of GERD with PPIs (esomeprazole, lansoprazole, omeprazole, pantoprazole, or rabeprazole) BID versus QD in a short-term setting (1 to 12 weeks), which reported relief of heartburn or healing of esophagitis as one of the primary outcomes. Articles were eligible for inclusion in this meta-analysis if they met the following criteria: (1) participants were diagnosed with GERD (RE or NERD or BE) based upon clinical features or upper endoscopy or 24 h esophageal pH and impedance monitoring or esophageal acid perfusion test; (2) participants were 18 years or older; (3) RCTs comparing PPIs BID to QD in the treatment of GERD; and (4) treatment duration for 1 to 12 weeks. The objective assessments of the efficacy of the treatment were the esophageal healing rates and the results of 24 h pH monitoring after the intervention. The subjective measurement was the relief rates of heartburn symptoms. The condition of the esophageal mucosa was graded according to the Los Angeles classification scale [11] or the modified 5-point Hetzel-Dent grading scale [12]. The severity of gastrointestinal symptoms was assessed by symptom scale [13–17]. Sustained resolution of heartburn was defined as seven consecutive days with a daily heartburn assessment of “none” [17].

### 2.3. Exclusion Criteria

Publications were excluded according to the following criteria: (1) not written in English; (2) not concerning a clinical question regarding human beings; (3) participants with extra esophageal complications; (4) missing or unclear data for final outcomes of interest; and (5) the duration lasted more than 3 months.

### 2.4. Study Selection

Two reviewers independently evaluated the titles and abstracts of the reports identified in the literature search for eligibility. Full-text versions of potentially relevant studies were obtained and double screened for eligibility. Disagreements were resolved by discussion.

### 2.5. Data Extraction

Data extraction was performed by two investigators. Data on publication status, trial design, patient characteristics, treatment regimens, methods, and results were extracted on a standardized form. All data were checked by a third investigator, and disagreements were resolved by a discussion.

### 2.6. Statistical Analysis

Appropriately 7 RCTs were included. All analyses were based on intention to treat (ITT), and a pooled estimate of odds ratio for PPIs once daily versus twice daily was calculated for our meta-analysis. All statistical analyses were done by using Review Manager Version 5.3 (The Cochrane Collaboration, Oxford, England) and Stata 12.0 software (StataCorp, College Station, TX, United States). The odds ratio of data was estimated by the Mantel-Haenszel *χ*^2^ method and when *P* values <0.05 were considered significant. Statistical heterogeneity between trials was evaluated using Cochrane *I*^2^ statistics test. Random effect modeling was applied if the *P* value for the test of heterogeneity was <0.10 by using the DerSimonian and Laird method; otherwise, we selected a fixed effect model. Possible publication bias was assessed by Egger's and Begg's funnel plots and when *P* values <0.05 indicated little publication bias.

## 3. Results

### 3.1. Search Results

**Figure 1:**
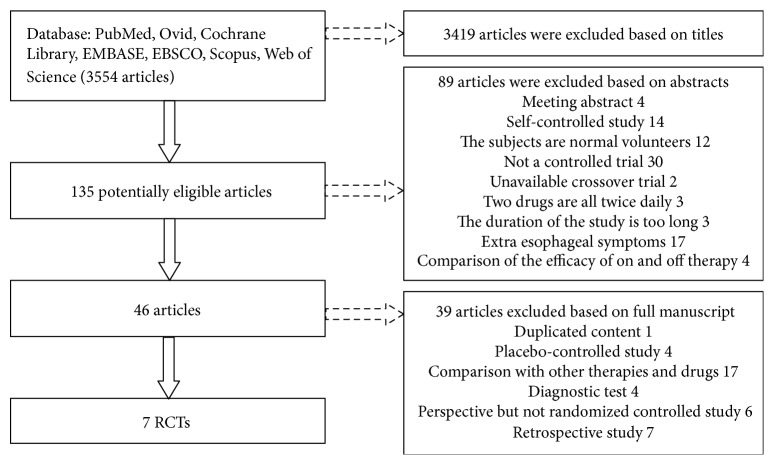


We initially identified 3554 publications using the search strategy, among which 3508 publications were excluded after examining the titles and abstracts. The remaining 46 articles were retrieved and evaluated in more details, of which 39 articles were excluded. Therefore, there were 7 studies included in the meta-analysis (Figure 1).

### 3.2. Study Characteristics

Table 1 depicts the baseline characteristics of the trials included in this review. The seven RCTs were published between 2000 and 2012 and included a total of 1710 patients. Three trials were conducted in European countries, two in the United States, one in Japan, and the last one in Taiwan (Table 1).

Two studies [13, 14] were conducted on patients with endoscopically confirmed reflux esophagitis (RE). And two studies [15, 16] were performed on subjects with refractory RE or GERD. One [18] study was conducted on patients with endoscopy, and 24 h esophageal pH or impedance test confirmed NERD patient. Another study [19] on patients with endoscopy-negative NERD and mild to moderate grade RE, which was diagnosed by endoscopy and 24 h esophageal pH monitoring, and the last study [17] on GERD patients confirmed by esophageal acid perfusion test.

Four trials [13, 15, 17, 18] compared the efficacy of standard doses of PPIs QD versus BID for GERD therapy. Three trials [14, 15, 19] estimated the efficacy of rabeprazole 20 mg QD versus rabeprazole 10 mg BID for GERD treatment. One trial [16] assessed the efficacy of standard dose of esomeprazole QD versus lansoprazole BID for GERD. In the United States and Europe, as well as many other countries around the world, rabeprazole 20 mg is considered as the standard dose for GERD therapy [20]. But in Japan, rabeprazole 10 mg is approved as a standard dose and 20 mg as a double dose [15]. We use the Western standard to judge the standard dose of rabeprazole for GERD therapy.

### 3.3. Symptom Relief

**Figure 2:**
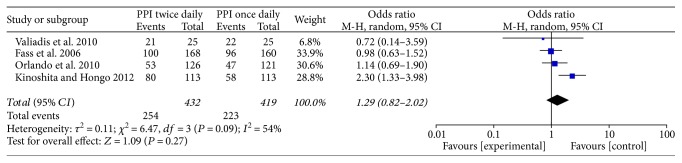


One trial [14] just used charts to show the trend of the relief of heartburn and regurgitation symptoms during the first 7 days, so we could not extract data to combine it with the results of other studies. In this study, the relief rates of symptoms between the rabeprazole 20 mg QD and 10 mg BID groups showed no significant differences at any time of the study period. Because most of the selected studies only providing data about the relief of heartburn symptoms, we combined the data about the relief rates of heartburn from four available studies [15–18]. All the four studies compared the efficacy of standard doses of PPIs QD versus BID in GERD patients. The pooled results did not have significant difference between the two groups (OR = 1.29, 95% CI: 0.82–2.02, *P* = 0.27) (Figure 2).

**Figure 3:**
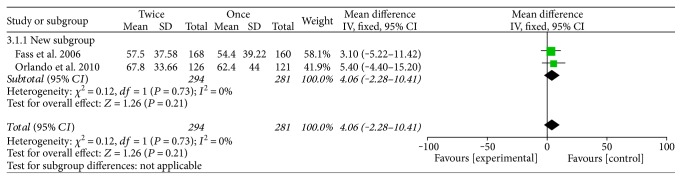


The sustained symptom relief rates at week 4 were reported in two studies [13, 17], which showed no significant difference between the groups of standard doses of PPIs BID versus QD (OR = 1.47, 95% CI: 1–2.16, *P* = 0.05). We also compared the results of heartburn-free days between the two groups [16, 17], but no significant difference was noted between them (*P* = 0.21) (Figure 3()). We assessed the symptom relief rates by dividing the patients into the refractory GERD or RE groups [15, 16] and the nonrefractory groups [17, 18], and the results of the two analyses both demonstrated no significant differences between the two groups (OR = 1.48, 95% CI: 0.64–3.4, *P* = 0.36; OR = 1.1, 95% CI: 0.67–1.78, *P* = 0.71), respectively.

### 3.4. Esophageal Healing

**Figure 4:**
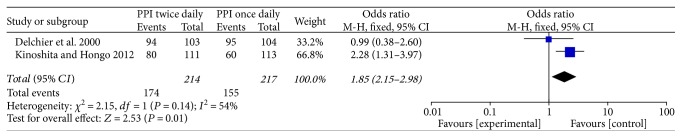


**Figure 5:**
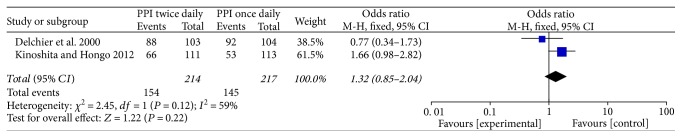


Only two studies [14, 15] compared the esophageal healing rates after 4 and 8 weeks' treatment. In comparing rates of healing of EE, we found that the differences between the rabeprazole 10 mg BID and 20 mg QD groups were significant after 8 weeks' treatment (OR = 1.85, 95% CI: 1.15–2.98, *P* = 0.01) (Figure 4). But the results had no significant difference after 4 weeks' treatment (OR = 1.32, 95% CI: 0.85–2.04, *P* = 0.22) (Figure 5).

There was an ARR of 10% and NNT of 10 after 8 weeks. According to the baseline endoscopic grading, one study [14] showed that the healing rates after 8 weeks' treatment between the two groups were similar in all the grades. The other trial [15] manifested that in patients with grade A or B RE, the healing rates were higher in the rabeprazole 10 mg BID (87.1%) and 20 mg BID (79.5%) groups as compared with those in the 20 mg QD (65.1%) group after 8 weeks, whereas the healing rates in patients with grade C or D RE were higher in the 20 mg BID (64.7%) group as compared with those in the 20 mg QD (25%) and 10 mg BID groups (35.3%) after 8 weeks' treatment. Based on the results of this study, rabeprazole 20 mg BID can achieve better mucosa healing than 20 mg QD after 8 weeks. However, both studies showed that there were no significant differences between the two groups after 4 weeks' treatment.

### 3.5. Twenty-Four-Hour pH Monitoring Results

**Figure 6:**
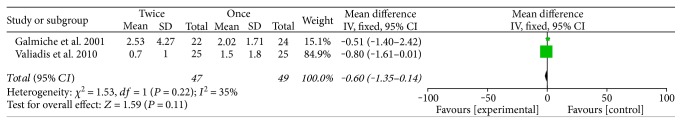


Two studies [18, 19] reported the results of pH and impedance monitoring. There were no statistically significant differences of the percentage of time that pH was maintained below 4 over 24 h between the rabeprazole 10 mg BID and 20 mg QD groups at baseline and at the end of the study in one trial [19]. In another study [18] which compared the efficacy of standard dose of esomeprazole QD with BID, the difference of percentage of total time pH < 4 at the end of study was statistically significant (*P* < 0.0001). When we combined the results of the two studies together, the difference between the BID and QD groups at the end of the study had no significant differences (*P* = 0.11) (Figure 6).

### 3.6. Adverse Events

**Figure 7:**
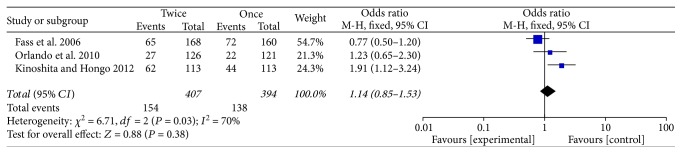


**Figure 8:**
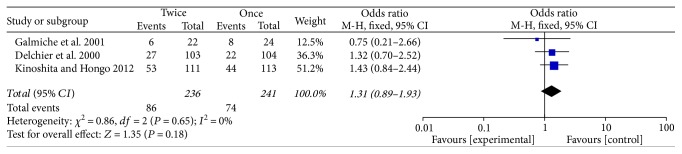


Five of the 7 trials reported adverse effects, including headache, osteoporosis, diarrhea, vomiting, flatulence, epigastric pain, and upper respiratory infection. Adverse events between the use of standard doses of PPI QD and BID groups were compared in three studies [15–17] (*P* = 0.38) (Figure 7) and between the rabeprazole 20 mg QD and 10 mg BID groups were compared in another three studies [14, 15, 19] (*P* = 0.18) (Figure 8). The results of the two comparisons both had no significant differences, respectively.

### 3.7. Risk of Bias Assessment

**Figure 9:**
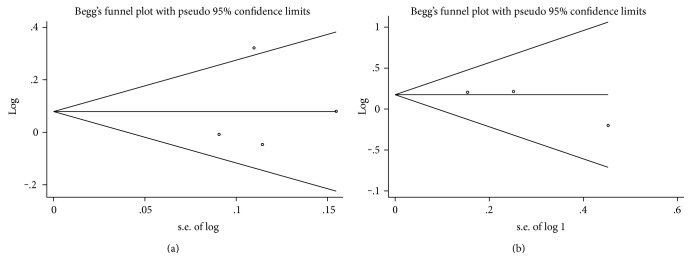


All of the trials clearly stated the method of randomization, concealment for allocation, and blinding of participants, except two studies [13, 18] did not illuminate whether it was double blind or not. A priori sample size calculation was performed in all the trials. Five trials [14–17, 19] were funded by pharmaceutical manufacturers. One trial [13] was supported by a grant from the National Scientific Council. Only one study [18] did not refer to supports from any associations or funds. No publication biases were detected in symptom response (Egger's test *P* = 0.834; Begg's test *P* = 1) (Figure 9(a)) and adverse event proportions (Egger's test *P* = 0.357; Begg's test *P* = 0.296) (Figure 9(b)).

## 4. Discussion

Previous study have reported that the healing rate for reflux esophagitis using standard PPI doses QD was 80–90% after 8 weeks [21]. Therapeutic trials have also shown that patients with NERD have a lower symptom response rate to PPIs once daily than patients with erosive esophagitis [22]. But no study had investigated the efficacy of the use of standard PPI doses or splitting standard PPI doses BID versus standard PPI doses QD for GERD treatment. In this systematic review and meta-analysis, we demonstrated that PPI BID therapy was in some extent more efficient than PPIs QD for the treatment of GERD. It did enhance the esophageal healing rates at week 8 when compared with QD PPI therapy.

A study in Germany [23] reported that GERD patients even those receiving routine clinical care and PPI therapy had an increase in time of work and decrease in work productivity. Another study [24] showed that persistent GERD symptoms despite PPI therapy had a significant and negative impact on both health-related quality of life (HR-QOL) and healthcare resource utilization. These findings reminded us to attach great importance to find appropriate options for symptomatic GERD patients in spite of taking routine PPIs. A recent guideline [25] for GERD recommended that in patients with partial response to PPIs QD, increasing the PPIs to BID or switching to a different PPI may provide additional symptom relief. And for patients who were refractory to standard doses of PPIs QD and PPIs BID may be a good choice. But the efficacy, doses, and duration of the treatment need to be further confirmed. Fujiwara et al. [26] proved that rabeprazole 10 mg BID improved GERD symptoms and adjusted sleep disturbances for patients with refractory GERD while receiving QD PPI treatment for 4–8 weeks. Fass et al. [27] have demonstrated that in patients who failed to standard dose of lansoprazole, administration of omeprazole 40 mg QD resulted in similar symptom compared with taking lansoprazole 30 mg BID after 6 weeks. Spechler et al. [28] showed that in BE patients, esomeprazole 40 mg BID was more effective than esomeprazole 40 mg QD, lansoprazole 30 mg QD, and BID in suppressing gastric acidity and well tolerated in short time. In another study [10], GERD patients and healthy volunteers were given omeprazole and lansoprazole BID for 7 days or 4 weeks. The 24 h intragastric pH monitoring was performed, which indicated that all these subgroups had unsatisfactory acid suppressive effects during night. A study performed on healthy pH-negative subjects manifested that the results of median pH values and pH > 4 holding time ratios were significantly higher in the rabeprazole 10 mg BID groups than in the 20 mg QD groups after 1 week [29]. From the above trials, we can see that the efficacies of the PPIs BID versus QD are not consistent.

In this study, we analyzed 7 RCTs to determine whether the therapeutic benefits of PPIs BID are superior to PPIs QD for GERD. We used subgroup analysis, according to the doses of the PPIs used in these studies. One comparison is conducted between the rabeprazole 10 mg BID and 20 mg QD groups; the other is performed between the standard doses of PPI QD and BID groups. We found no significant difference regarding sustained symptom relief at week 4, symptom relief rates, and 24 h pH monitoring results. However, the endoscopic response after 8 weeks seems to be better in the BID groups than in the QD groups. With respect to adverse events, no significant differences are noted between the two groups.

There are some limitations in our meta-analysis to be considered. Firstly, the number of studies included in this analysis is limited. Only two studies were combined to compare the efficacy. This to some extent leads to the heterogeneity of the results. Secondly, the PPIs used in these 7 trials were not identical, so we divided them into subgroups according to the doses of PPIs and keep them as consistent as possible among different studies. There are different PPIs available for the treatment of GERD, including omeprazole, lansoprazole, rabeprazole, pantoprazole, esomeprazole, and others, although the impacts of different PPIs were not significantly different on symptom relief in a previous meta-analysis [30]. Thirdly, we cannot rule out the possible interference of treatment duration to the results. Although only studies with duration between 1 week and 12 weeks were selected, they have different treatment duration. Fourthly, the baseline characteristics of these studies may have slight differences. The average age of the subjects in one study [15] was greater than other studies. Two studies [15, 16] recruited patients with refractory RE or GERD, while the subjects of other studies were all GRED patients. We performed subgroup analysis to resolve this problem. One study [13] selected the overweight and obese subjects specially, but the weights of them seemed similar to the subjects of other studies due to the racial differences. Finally, when we analyzed the results of the 24 h pH monitoring, one study [18] showed significant difference between the esomeprazole 40 mg QD and BID groups, but another study [19] reported no significant difference between rabeprazole 10 mg BID and 20 mg QD. The combined results did not present significant difference. This may be attributed to the reasons that the study duration in the latter trial was insufficient compared with that in the prior study. Perhaps, the results may be different when the study duration was prolonged. To overcome all the above limitations and drawbacks that some studies failed to find significant symptom improvement in the peer-reviewed literature [31], in later work, well-designed RCTs are needed to be conducted with a larger quantity of participants to more effectively determine the efficacy and safety profiles of PPI BID treatment for GERD.

## 5. Conclusion

In summary, patients with GERD respond to PPI BID treatment, which may improve endoscopic healing rates at week 8 more effectively than PPIs QD. However, there were no significant differences in symptom relief, sustained symptom relief at week 4, endoscopic response after 4 weeks, and 24 h pH monitoring results after treatment between them. Adverse effects of PPI BID and PPI QD therapy may be compared. Whether PPIs BID is indeed therapeutically more efficient than PPIs OD and whether the specific optimal doses and duration of PPI treatment for GERD require future trials need to be identified.

## Figures and Tables

**Table 1:** Baseline characteristics of the 7 randomized controlled trials included in this meta-analysis comparing the treatment effects of PPIs once daily versus twice daily for GERD patients.

Author	Country	Participants	Duration	Age (mean or mean ± SD, y)	Male (%)	Weight (kg) or BMI	Intervention
Orlando et al. 2010	USA	368	4 weeks	43.7	46.3%	29.2	Esomeprazole 20 mg QD
				43.9	42.1%	29.8	Esomeprazole 40 mg QD
				46.9	38.1%	30.5	Esomeprazole 40 mg BID

Delchier et al. 2000	Europe	310	8 weeks	55 ± 15.7	55%	N	Rabeprazole 20 mg QD
				52 ± 14.3	70%	N	Rabeprazole 10 mg BID
				53 ± 15.1	61%	N	Omeprazole 20 mg QD

Fass et al. 2006	USA	328	8 weeks	49 ± 12.5	40.6%	86.9 ± 19.7	Esomeprazole 40 mg QD
				48.3 ± 13.6	45.8%	87.5 ± 19.2	Lansoprazole 30 mg BID

Kinoshita and Hongo 2012	Japan	337	8 weeks	64.5 ± 13.9	51.8%	24.14 ± 3.61	Rabeprazole 20 mg QD
				65.5 ± 13.3	46.8%	24.89 ± 3.68	Rabeprazole 10 mg BID
				66.6 ± 13.8	44.1%	24.32 ± 4.04	Rabeprazole 20 mg BID

Chen et al. 2010	Taiwan	200	8 weeks	42.6	63.36%	27.9 ± 2	Pantoprazole 40 mg QD
				43.1	56.43%	28.2 ± 2.1	Pantoprazole 40 mg BID

Vasiliadis et al. 2010	Greece	75	30 days	40.96 ± 11.98	70.8%	26.6 ± 0.97	Esomeprazole 40 mg BID
				44.17 ± 11.18	75%	27.2 ± 0.87	Esomeprazole 40 mg QD
				42.68 ± 11.34	72%	26.8 ± 1.17	Esomeprazole 40 mg QOD

Galmiche et al. 2001	France	92	1 week	42.1 ± 14.3	63.64%	N	Rabeprazole 10 mg BID
				39.4 ± 13.8	66.67%	N	Rabeprazole 20 mg QD
				41.2 ± 14.2	73.91%	N	Omeprazole 20 mg QD
				43.6 ± 11.3	52.17%	N	Placebo
